# Association between Maternal Dietary Diversity and Low Birth Weight in Central India: A Case-Control Study

**DOI:** 10.1155/2021/6667608

**Published:** 2021-05-30

**Authors:** Shantanu Sharma, Sonali Maheshwari, Sunil Mehra

**Affiliations:** ^1^Lund University, Lund, Sweden; ^2^MAMTA Health Institute for Mother and Child, Delhi, India

## Abstract

Low birth weight (LBW) is one of the major public health challenges in India. LBW etiology is multifactorial and linked to multiple determinants, including maternal undernutrition and sociodemographic characteristics. The objective of the present endeavor was to assess how maternal dietary diversity and other sociodemographic factors among marginalized populations are associated with the incidence of LBW. The study was a part of the community-based intervention that aimed to improve maternal and child health in the Morena district of Madhya Pradesh, a state in central India. In this case-control study, cases were defined as mothers with an LBW child (<2500 grams) and controls as mothers without an LBW child. A quantitative survey was done with women of reproductive age, having at least one child aged 0–24 months. We calculated the dietary diversity based on the number of food groups consumed during pregnancy by women on a daily basis. Stepwise logistic regression models were built to test for associations between sociodemographic and dietary diversity variables and LBW incidence. There were 157 mothers with and 214 without an LBW child. Women's diets mainly consisted of grains, such as wheat, rice, maize, and roots and tubers. Eggs and meat were consumed by less than 1% of the women. There were 20% lesser chances of an LBW child with increasing maternal dietary diversity scores (odds ratio: 0.79; 95% CI: 0.65, 0.96). The poor maternal diet quality during pregnancy may result in adverse birth outcomes with long-term consequences in a child.

## 1. Introduction

Low birth weight (LBW), defined as birth weight less than 2500 grams, is associated with increased child morbidity and mortality [1]. Globally 20 million births are LBW in a year, 90% of which occur in low- and middle-income countries (LMIC) [2]. India alone accounts for 40% of all LBW babies born in LMIC [3]. Although the prevalence of LBW has significantly declined from 20.4% to 16.4% in the last decade, it continues to be one of the major public health challenges in India [4]. The percentage of LBW among newborns varies markedly across geographies in India, with states like Odisha, Assam, and Madhya Pradesh, reporting the prevalence around 14–18% [5].

Multiple risk factors associated with LBW include maternal age, socioeconomic characteristics, and nutritional status before and during pregnancy [6]. Maternal undernutrition, including macronutrient and micronutrient deficiencies, is causally linked with LBW and two of its underlying causes, including preterm births and small for gestational age [7]. Maternal undernutrition is widely prevalent in the country, especially among marginalized populations. According to the national reports, 23% of women aged 15–49 were underweight and 50% anaemic in India [8]. In addition, some studies conducted in the country showed that around 30% of pregnant women had copper deficiency [9, 10], and 65% of pregnant women had zinc deficiency [3].

Nutrition is key to accomplish sustainable development goals (SDG) 2030 and improved maternal and child nutrition is a crucial strategy in this regard for countries to achieve SDG 2030 [11]. In its attempt to improve maternal nutrition status in the country and prevent its transgenerational impacts, the government of India is implementing multiple programs. These programs include iron folic acid (IFA) and calcium supplementation during and 6 months after pregnancy, food supplementation through Integrated Child Development Centres (*Anganwadi Centres*), conditional cash transfer scheme (*Pradhan Mantri Matru Vandana Yojna*), and quality antenatal care, including nutritional status monitoring and nutrition-related counselling [12]. However, considerable gaps exist in effective implementation of these programs; as a result, only 18% of pregnant women meet recommendations of dietary diversity (≥5 food groups), 2.4% for IFA intake (180 tablets), and 1.1% for calcium tablets' consumption (180 tablets) [13]. Furthermore, on average, Indian women gain 7 kg weight during pregnancy against the recommendation of 10–12 kg [13].

Considering the need to deliver and monitor the effectiveness of nutrition-focused interventions in the country, emerging evidence suggests the use of a simple assessment tool to account for the complex behavior of food consumption during pregnancy. Dietary diversity is an effective tool to understand the relationship between maternal nutrition adequacy during pregnancy and birth outcomes [6]. Dietary diversity, defined as the number of individual food items or food groups consumed over a given period of time, can be used as a reference to improve dietary quality and micronutrient status during pregnancy [6, 14].

*Parivartan* was an initiative by the MAMTA Health Institute for Mother and Child to improve maternal and child health outcomes among marginalized populations in Morena district of Madhya Pradesh, a state in central India. To improve maternal and child nutrition status in the district, *Parivartan* aimed to understand maternal undernutrition in the local context and how did it affect the birth outcomes along with other sociodemographic determinants. Hence, in the context of this implementation research effort, our paper assessed how maternal dietary diversity and other sociodemographic factors among marginalized populations were associated with the incidence of LBW.

## 2. Materials and Methods

### 2.1. Study Design and Study Population

It was a community-based case-control study. Quantitative data were obtained from the survey conducted in 2018 as a part of the baseline study with women of reproductive age having at least one child aged 0–24 months. Cases were defined as mothers with an LBW child (<2500 grams) and controls as mothers without an LBW child. The survey was carried out in 2 blocks from Morena district of Madhya Pradesh. The Morena district, situated in the northern part of the state, has a population of 1,965,970 and a sex ratio of 839 females per 1000 males. In the district, 30% of women aged 15–49 have Body Mass Index (BMI) less than 18.5 Kg/m^2^ while 55% of pregnant women aged 15–49 are anaemic [8].

### 2.2. Sample Size and Sampling

The sample size was calculated at 105 each for cases and controls by using the following formula:

(1) $$\begin{array}{c} n=\left(\frac{r+1}{r}\right)\frac{\left(\bar{p}\right)\left(1-\bar{p}\right){\left(Z_{\beta }+Z_{\alpha /2}\right)}^{2}}{{\left(p_{1}-p_{2}\right)}^{2}}, \end{array}$$

where *r* = 1 (equal number of cases and controls). $\bar{p}$ which is a measure of variability =(p1+p2)/2. *p*_1_ which is the proportion exposed in the control group (low maternal dietary diversity in a normal birth weight baby) = 0.46 [15]. p_2_ which is the proportion exposed in the case group (low maternal dietary diversity in an LBW baby) = 0.66 [15]. For 80% power, *Z*_*β*_ = .84. For 0.05 significance level, *Z*_*α*_ = 1.96.

A two-stage sampling technique was adopted to obtain an appropriate sample for the study. In the first stage, 12 villages (primary sampling unity, PSU) were selected from the district using Probability Proportional to Size (PPS) technique. In the second stage, from each village, 32 eligible respondents were randomly selected from a list. The list of the eligible respondents was collected from the community health workers, and final subjects were identified randomly using computer-generated random numbers.

### 2.3. Study Tool

A structured quantitative questionnaire was used to collect information on sociodemographic characteristics, food groups consumed during pregnancy, and antenatal history. The sociodemographic indicators included the following: age, age at marriage, education status (illiterate/primary/upper primary/secondary/higher secondary and above), religion (Hindus/Muslim), caste (scheduled caste/scheduled tribe/other special class), socioeconomic status (above or below the poverty line), and family monthly income (Indian Rupees, INR). Similarly, questions related to antenatal history included the place of delivery, weight gain during pregnancy, received any financial aid from the government, registration of the pregnancy, received antenatal care by a skilled birth attendant, the number of antenatal check-ups done (<4 or ≥4 visits), and the number of IFA and calcium tablets consumed. The dietary information during pregnancy was obtained using a food frequency questionnaire (FFQ). The prevalidated FFQ included the consumption of food items from 10 food groups as outlined by the Food and Agriculture Organization (FAO) [6, 14].

The dietary diversity was calculated based on the number of food groups consumed during pregnancy by women on a daily basis. The ten food groups used in the FFQ were (a) grains, white roots, and tubers; (b) pulses, including beans and lentils; (c) nuts and seeds; (d) dairy (milk and milk products); (e) meat, poultry, and fish; (f) eggs; (g) dark green leafy vegetables; (h) other vitamin A-rich fruits and vegetables; (i) other fruits; (j) other vegetables [14]. We assigned a score of 1 to each food group if the subject had consumed it on a daily basis or 0 if not. The scores of the ten food groups were summed up to calculate the total dietary diversity score of women.

### 2.4. Dependent and Independent Variables

The dependent variable was the birth outcome (LBW incidence), categorized into mothers who had an LBW child (<2500 grams) and mothers without an LBW child (≥2500 grams). The independent variables included all the sociodemographic determinants, dietary diversity, and antenatal care practices.

### 2.5. Ethics Consideration

Written informed consent was obtained from the participants after explaining the purpose, risk, and expected outcomes of the study. Ethical approval was obtained from the Institutional Review Board (IRB) of MAMTA Health Institute of Mother and Child, New Delhi.

### 2.6. Data Collection and Analysis

Data were collected by a team of six investigators in the local language (Hindi). We used descriptive analysis to report the study population characteristics. Bivariate analysis was performed to test for associations between independent variables and birth outcomes (LBW incidence). Thereafter, stepwise logistic regression models were built, first with all the sociodemographic determinants and dietary diversity, followed by sociodemographic variables, dietary diversity, and variables of antenatal care practices. Odds ratio (Beta coefficients) and 95% confidence intervals were estimated for logistic regression. All the analysis was performed using IBM SPSS Statistics for Windows version 25.0 (IBM Corp., Armonk, NY, USA).

## 3. Results

There were 157 mothers who had an LBW child and 214 mothers without an LBW child (Table 1). The mean age of the mother in both groups at the time of the interview was 25 (standard deviation: 3.7) years. Nearly half of the women were illiterate or educated up to primary level among both groups. More than 90% of women delivered in institutions, as shown in Table 1. Mothers with an LBW child gained 6.6 kg during pregnancy compared to 7.5 kg by mothers without an LBW child. On average, women with an LBW child consumed 60 IFA tablets during pregnancy, whereas women without an LBW child consumed 90 IFA tablets during pregnancy.

**Figure 1:**
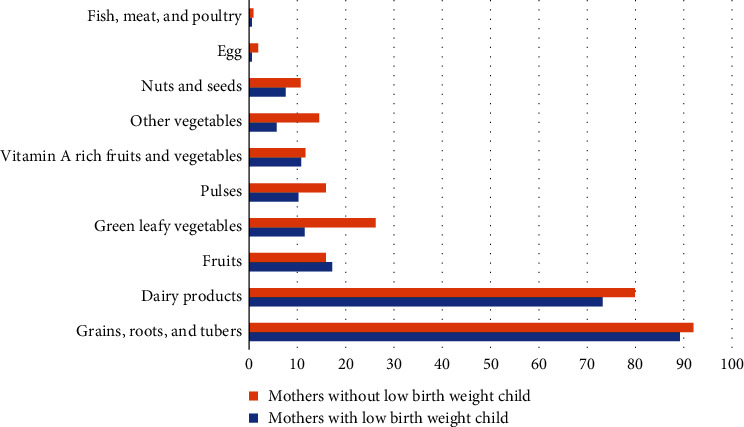
Distribution of the consumption rate of ten food groups between mothers with and without low birth weight child on a daily basis.

Women's diets mainly consisted of grains, such as wheat, rice, maize, and roots and tubers (Figure 1). Figure 1 illustrates that grains, roots, and tubers (wheat, rice, maize, etc.) are the most commonly consumed food group among women followed by dairy products. The consumption of eggs and nonvegetarian food items is the least among all the food groups. The consumption rate is nearly similar in both groups (low birth or normal birth weight) except for vegetables, that is, green leafy and other vegetables.

The consumption of dairy products was common among two-thirds of women in both groups. Eggs and meat were consumed by less than 1% of the women. Maternal dietary diversity was negatively associated with the incidence of LBW (Table 2). There were 20% lesser chances of an LBW child with increasing maternal dietary diversity scores. In the unadjusted regression analysis, increased weight gains during pregnancy had a 12% lower probability, and less than 4 ANC visits had 1.7 times higher odds of having an LBW child. However, such associations turned insignificant in the adjusted regression model. Similarly, women who belonged to scheduled castes/tribes had 1.8 times higher odds of an LBW child than those from other special classes.

## 4. Discussion

The study contributed to emerging evidence of maternal dietary practices during pregnancy on birth outcomes (LBW). We found 20% decreased odds of an LBW child among mothers who had a higher dietary diversity. The association was significant after adjustments for potential confounders like maternal educational status, socioeconomic status, early marriage, and antenatal care.

Situated in the centre of India, Madhya Pradesh is a state with very high maternal and neonatal mortality rates compared to the rest of India [5, 8]. Similarly, Morena is one of the poor performing (backward) districts of Madhya Pradesh, with most of its health indicators much below the state average [8]. According to the national survey, 46% of women are illiterate, 30% are married off early (<18 years), only 36% completed four or more antenatal visits, and only 15% took 100 IFA tablets in the district during pregnancy. Our study reports similar findings of high early marriage (19–21%), illiteracy or education up to primary level (47–53%), and low coverage of 4 or more antenatal visits (44–57%) among women [8, 16]. The majority of the population in the district belongs to rural areas and backward classes like scheduled caste/tribe [17]. Previous studies have highlighted that Madhya Pradesh has wide spatial differences in the maternal and child health status and utilization of maternal health services. The uptake of services is far better in districts with higher levels of urbanization and those located in the industrial region [17, 18].

Unlike other studies, sociodemographic characteristics like low maternal educational status and early marriage were not found to be associated with LBW babies in our study [4, 19, 20]. Early marriages followed by teenage pregnancy carry a higher risk of complicated pregnancies, including LBW, premature births, and need for neonatal intensive care [21]. India has the highest number of child brides in the world and states like Bihar, Jharkhand, and Madhya Pradesh have a high rate of teenage pregnancies [21, 22]. It is to note that these teenage mothers are physiologically weak and cognitively immature to identify their role as responsible mothers [22]. So there is a need to focus on some constructive ways like health literacy through peer-led interventions or community engagement models to reduce child marriages, delay first pregnancy, and provide good quality antenatal care to teenage mothers to have better mother and child health.

It is widely acknowledged that weight gain, anemia, IFA, and calcium consumption during pregnancy influence the incidence of LBW [23, 24]. We found a relatively higher proportion of LBW babies among women who had lower consumption of IFA and calcium tablets. A substantial proportion of the population in India is anaemic, and, in fact, India has one of the highest anemia prevalence rates in the world [25]. Despite the country's anemia control program of IFA supplementation running for decades, anemia continues to be a public health challenge such that one in two pregnant women has hemoglobin less than 11 gm% [8]. Multiple reasons deterrent to successful operationalization of IFA supplementation include poor supply and logistics management of the tablets, ineffective counselling by frontline workers, lack of awareness and negative perceptions, or social norms related to its side effects among women [13, 26].

In the move to accelerate the reduction in anemia prevalence in the country, the government of India launched the “Anemia Free India” program in 2018. The program aims to target 30 million pregnant women through 6 different interventions, including prophylactic IFA supplementation, behavior change actions, deworming, point of care testing for anemia, addressing nonnutritional causes of anemia like malaria, hemoglobinopathies, and provision of iron-fortified foods in government programs [27]. Other studies demonstrated that underweight women or those who gained less weight during pregnancy are at a higher risk of delivering an LBW child [28]. Though the national guideline recommends an average weight gain of 10–12 kg during pregnancy, there are no recommendations on the weight gain based on the prepregnancy weight [29]. We suggest preconception counselling for women emphasizing the need for adequate food intake and weight gain during pregnancy.

Our study findings corroborate with other studies that women who made the recommended number of antenatal visits (four or more) had fewer odds of having a baby with LBW than women who had fewer visits [30]. Frequent antenatal check-ups offer vigilant monitoring and tracking of pregnancies, which could help identify complications and risks early. We argue that not only the frequent antenatal check-ups but also the quality of care during visits are equally important. The World Health Organization recommends a minimum of 4 antenatal care visits appropriate in content, which suggests monitoring of weight, height, urine and blood analysis, tetanus vaccination, and prescription of IFA and other dietary supplements during these visits [31]. Similar to the previous studies, we found higher consumption of grains, roots, and tubers and lower consumption of eggs, fish, poultry, and meat among women than other food groups. It is widely known that vegetarian diet rich in starch/carbohydrates is a predominant dietary pattern among Indian women [32, 33]. Notably, dairy products' consumption increases during pregnancy and postpregnancy among women. This differential intake of food groups is linked to religious or social norms and beliefs prevalent in the society and geographical variations that determine their availability [34, 35].

There is empirical evidence of the association between maternal undernutrition and poor dietary intake with LBW [7]. However, we lacked evidence on the influence of dietary diversity (a proxy indicator of dietary quality) on birth outcomes from the previous studies. Our study is one of the few studies that have calculated maternal diversity to assess the risk of one of the important public health challenges, that is, LBW, in LMIC. In our study, mean maternal dietary diversity was very low (2.2–2.7) compared to the recommendations (≥5). A huge population in India is food insecure, largely due to poverty [36]. Despite food being a basic human right, one in nine people in the world experience chronic hunger [37]. Women from food-insecure households have a low diverse diet and are less likely to consume animal source foods like eggs, dairy products, meat, and so on, micronutrient-dense plant-based foods like nuts and legumes, and vitamin A-rich fruits and vegetables [36]. The inadequate diet and poor quality “less diverse” diet can result in chronic undernutrition and multiple nutrient deficiencies over generations. The plausible mechanisms of the influence of maternal undernutrition on birth outcomes include limited nutrient availability for transfer to the fetus and increased risks of infections and maternal morbidity, thereby leading to fetal inflammation and epigenetic programming [38].

Our results are congruent with another study conducted in Uttar Pradesh, a state with high maternal and newborn mortality [15]. The study reported that women with low maternal dietary diversity had a significantly higher proportion of LBW babies compared to those in the medium to high dietary diversity categories. Additionally, the study found that low maternal education and economic status were significantly associated with poor dietary diversity among participants. Furthermore, factors such as maternal smoking, alcohol consumption, malaria, and HIV infections have been highlighted to influence birth outcomes in other studies [15, 20].

Studies have entrusted upon dietary diversity as a promising measurement tool but recommend researchers to carefully explore how it can be operationalized and used to determine the purposes it could serve [39]. It is widely used in large studies and has the advantage of being inexpensive for assessing diet quality.

### 4.1. Limitations of the Study

The study results should be interpreted in view of certain limitations. Dietary diversity in pregnancy was assessed using an FFQ method during the postpregnancy period, which may add a recall bias. The case-control study design was apt in the current situation because the long-term follow-up of the cohort is not only time-consuming but cost-intensive as well.

## 5. Conclusions

Our study used a case-control study design to show that women with high maternal dietary diversity living in the rural areas of Morena district of Madhya Pradesh, India, had 0.8 times the odds of having an LBW baby. Overall, the maternal dietary diversity was low in both groups, mothers with or without an LBW child. A multidimensional approach is required to improve the diet quality of women during pregnancy, including awareness generation to eat a diversified diet, improving access to poverty eradication and food security programs, and increasing the uptake of IFA and calcium supplementation.

## Figures and Tables

**Table 1:** Sociodemographic characteristics, maternal dietary diversity, and antenatal history of mothers with and without low birth weight child.

Variables	Mothers with a low birth weight child (*n* = 157) N (%)	Mothers without a low birth weight child (*n* = 214) N (%)
Age (years) of mother at the time of the interview, mean (±SD)	25.1 (±3.7)	25.0 (±3.4)
Missing (*n*)	3	1

Early marriage		
Yes	32 (21.0)	39 (19.0)
No	121 (79.0)	165 (81.0)
Missing (*n*)	4	10

Education status		
Illiterate or educated up to primary level	73 (47.1)	112 (53.1)
Upper primary	53 (34.2)	52 (24.6)
Secondary and above	29 (18.7)	47 (22.3)
Missing (*n*)	2	3

Religion		
Hindus	151 (96.2)	208 (97.7)
Muslims	6 (3.8)	5 (2.3)
Missing (*n*)	0	1

Caste		
Scheduled caste/tribe	48 (31.2)	47 (22.4)
Other special classes	106 (68.8)	163 (77.6)
Missing (*n*)	3	4

Socioeconomic status		
Above the poverty line	111 (74.0)	56 (69.3)
Below the poverty line	39 (26.0)	62 (30.7)
Missing (*n*)	7	12

Monthly income (INR), median (interquartile range)	5000 (3000–8000)	5000 (3000–8000)
Missing (*n*)	10	9

Maternal dietary diversity^⁑^		
Mean (±SD)	2.2 (±1.3)	2.7 (±1.7)

Place of delivery		
Government hospital	145 (94.8)	195 (91.5)
Private hospital	6 (3.9)	18 (8.5)
Home	2 (1.3)	0
Missing	4	1

Weight gain during pregnancy^⁑^ (kg), mean (±SD)	6.6 (±2.4)	7.5 (±2.7)
Missing	10	16

Any financial aid from the government		
Yes	97 (61.8)	135 (63.7)
No	60 (38.2)	77 (36.7)
Missing	0	2

Registered pregnancy		
Yes	144 (92.9)	203 (94.9)
No	11 (7.1)	11 (5.1)
Missing	2	0

Antenatal care by skilled birth attendant		
Yes	67 (45.9)	75 (36.9)
No	79 (54.1)	128 (63.1)
Missing	11	11

Number of ANC visits^§^		
<4 visits	81 (56.3)	83 (42.6)
4 or more	63 (43.8)	112 (57.4)
Missing	13	19

Number of IFA tablets^§^ consumed		
Median (IQR)	60 (30–100)	90 (30–180)
Missing	30	39

Number of calcium tablets^§^ consumed		
Median (IQR)	30 (12–85)	45 (25–92)
Missing	77	92

ANC: antenatal care; INR: Indian Rupees; IFA: iron folic acid tablets; IQR: interquartile range; SD: standard deviation. ^⁑^Association significant at *p* < 0.01. ^§^Association significant at *p* < 0.05.

**Table 2:** Logistic regression analysis showing stepwise regression for low birth weight with respect to selected background characteristics in Madhya Pradesh, India.

Variables	Mothers with a low birth weight child^§^		
	Unadjusted ß (95% CI), *p* value	Adjusted model I. *ß* (95% CI), *p* value	Adjusted model II. *ß* (95% CI), *p* value
Maternal dietary diversity	**0.81 (0.69–0.94)** ^∗∗^	**0.74 (0.61–0.88)** ^∗∗∗^	**0.79 (0.65–0.96)** ^∗^
Early marriage			
Yes	1.12 (0.66–1.88)	1.12 (0.62–2.01)	0.88 (0.44–1.75)
No	*Reference*	*Reference*	*Reference*
Education status			
Illiterate or educated up to primary level	1.05 (0.61–1.83)	0.81 (0.43–1.50)	0.80 (0.40–1.57)
Upper primary	1.65 (0.90–3.01)	1.54 (0.79–3.00)	1.57 (0.75–3.27)
Secondary and above	*Reference*	*Reference*	*Reference*
Caste			
Scheduled caste/tribe	1.57 (0.98–2.51)	**1.71 (1.01–2.91)** ^∗^	**1.83 (1.02–3.27)** ^∗^
Other special classes	*Reference*	*Reference*	*Reference*
Socioeconomic status			
Above the poverty line	1.26 (0.78–2.02)	**1.76 (1.03–3.02)** ^∗^	1.58 (0.88–2.84)
Below the poverty line	*Reference*	*Reference*	*Reference*
Age (years)	1.00 (0.95–1.06)	1.01 (0.94–1.07)	1.01 (0.93–1.09)
Weight gain during pregnancy (grams)	**0.88 (0.81–0.96)** ^∗∗^	—	0.93 (0.84–1.03)
Any financial aid from the government		—	
Yes	0.92 (0.60–1.41)		0.97 (0.55–1.70)
No	*Reference*		*Reference*
Antenatal care by skilled attendant		—	
Yes	1.44 (0.94–2.23)		0.93 (0.55–1.58)
No	*Reference*		*Reference*
Number of ANC visits		—	
Less than 4 visits	**1.73 (1.12**–**2.68)**^∗^		1.67 (0.98–2.87)
≥4 visits	*Reference*		*Reference*

ANC: antenatal care; CI: confidence interval; LBW: low birth weight; Β is the odds ratio. ^§^Mothers without a low birth weight child were the reference category. ^∗^Significant at *p* < 0.05. ^∗∗^Significant at *p* < 0.01. ^∗∗∗^Significant at *p*=0.001. Adjusted model 1: sociodemographic characteristics; adjusted model II: model I + antenatal care practices.

## Data Availability

The data related to this manuscript are not provided due to institutional policy but can be made available upon personal request.
